# Evaluation of Androgen Receptor in Relation to Estrogen Receptor (AR/ER) and Progesterone Receptor (AR/PgR): A New Must in Breast Cancer?

**DOI:** 10.1155/2019/1393505

**Published:** 2019-04-14

**Authors:** Giuseppe Bronte, Andrea Rocca, Sara Ravaioli, Emanuela Scarpi, Massimiliano Bonafè, Maurizio Puccetti, Roberta Maltoni, Daniele Andreis, Giovanni Martinelli, Sara Bravaccini

**Affiliations:** ^1^Istituto Scientifico Romagnolo per lo Studio e la Cura dei Tumori (IRST) IRCCS, Meldola, Italy; ^2^Department of Experimental, Diagnostic and Specialty Medicine, Alma Mater Studiorum, University of Bologna, Bologna, Italy; ^3^AUSL (Azienda Unità Sanitaria Locale) Imola, Imola, Italy

## Abstract

Steroid nuclear receptors are known to be involved in the regulation of epithelial-mesenchymal transition process with important roles in invasion and metastasis initiation. Androgen receptor (AR) has been extensively studied, but its role in relation to breast cancer patient prognosis remains to be clarified. AR/ER ratio has been reported to be an unfavorable prognostic marker in early primary breast cancer, but its role in the patients with advanced disease has to be cleared. We retrospectively analyzed ER, PgR, and AR expression on a case series of 159 specimens of primary BC samples by using immunohistochemistry and 89 patients of these had luminal tumors for which AR and ER expression and survival data were available. For twenty-four patients both primary and metastatic tumors were available. A significantly shorter overall survival was observed in primary tumors with AR/PgR ratio ≥ 1.54 (HR = 2.27; 95% CI 1.30-3.97;* p* = 0.004). Similarly OS was significantly shorter when ER/PgR ratio ≥2 in primary tumors (HR = 1.89; 95% CI 1.10-3.24;* p* = 0.021). The analysis of the 24 patients who had biomarker determinations both in primary tumors and metastasis showed a better OS when AR/ER ratio in the metastasis was ≥ 0.90 (*p* = 0.022). Patients with a high AR/ER ratio in primary tumor that remained high in the metastasis had better prognosis in terms of OS (*p* = 0.011). Despite we suggested that the ratios AR/ER and AR/PgR could be used to identify patients with different prognosis, their real value needs to be better clarified in different BC settings through prospective studies.

## 1. Introduction

Steroid nuclear receptors are known to have an important role in the regulation of epithelial-mesenchymal transition (EMT) process and in the tumor progression [1].

Beyond specific EMT-inducing factors such as Snail and Slug, a “*multiverse*” of factors, including hormone receptor signaling pathways are involved in metastasis establishment regulating cell plasticity and motility [2]. Given that EMT transcription studies in breast cancer mainly focus on triple negative breast cancer (BC) subtype BRCA1 addicted, more information is needed in luminal cancers [3].

Androgen receptor (AR) may have EMT promoting effects through the suppression of E-cadherin promoter activated by dihydrotestosterone. This is in contrast with the fact that AR expression is associated with a good prognosis suggesting a tumor and EMT-suppressing effects [1].

The possibility that a receptor for androgen is expressed in BC is fascinating given that the tumor is predominantly estrogen-dependent.

Although the AR has been extensively studied, its role in this malignancy remains to be clarified as well as its relation with the other steroid nuclear receptor involved in BC biology, such as estrogen receptor (ER) or progesterone receptor (PgR). Researchers are currently trying to understand whether AR interferes with ER or PgR activity. AR is already a therapeutic target, and the availability of selective AR inhibitors (e.g., bicalutamide, enzalutamide, apalutamide) approved for the treatment of prostate cancer has opened up the possibility of their use in BC patients whose tumors express AR. However, things are not as simple as they seem as AR appears to have different functions according to the BC subtype, e.g., ER positive or triple negative BC. In ER-negative BC, studies on the prognostic effect of AR expression yielded conflicting results [4, 5] even though AR can predict response to AR inhibitors [6]. Conversely, patients with ER positive and AR positive have a better outcome than those with ER positive and AR-negative disease [4, 7]. This has been attributed to the competition between AR and ER at the level of estrogen response elements (EREs) and consequent impairment of ER-dependent gene transcription [8].

A few years ago Cochrane et al. reported a worse prognosis when AR levels were high and ER levels were low, which resulted in a resistance to tamoxifen. The authors defined the relationship between AR and ER expression as the AR/ER ratio. They had been prompted to perform this study by the observation that, while BC is well differentiated and more indolent when AR is expressed, AR overexpression leads to tamoxifen resistance in* in vitro* and* in vivo* studies. This study can be considered a seminal work because the authors introduce the concept of the AR/ER ratio for the first time. This new parameter could be useful in ER positive BC as the majority of these tumors (84-91%) are AR positive. Hence, the simple evaluation of AR expression may not be sufficient to further subdivide the ER positive subset into different prognostic groups. Cochrane et al. identified 2.0 as the best AR/ER cut-off value to classify patients according to disease-free survival (DFS). In their study of around 200 patients with primary BC, an AR/ER ratio ≥ 2.0 identified a subgroup with a four-fold higher risk of failure during adjuvant tamoxifen [9].

More recently, Rangel et al. explored the usefulness of the AR/ER ratio in a larger population of ER+/HER2- BC patients. A higher AR/ER ratio was associated with unfavorable features (e.g., larger primary tumor, higher nodal status, higher histological grading). Like Cochrane et al., the authors identified 2.0 as the best cut-off to distinguish between prognostic cohorts. Thus, an AR/ER ≥ 2.0 predicted poor survival in primary BC patients in terms of both disease-free interval (five-fold higher risk of relapse) and disease-specific survival (> eight-fold higher risk of relapse) [10].

The results from the above 2 studies in which the AR/ER ratio was used to estimate prognosis in patients with primary luminal BC (i.e., ER+/HER2-) raises the question as to whether this new parameter will become mandatory for prognostic classification in this BC subset. We previously showed that the AR/PgR ratio, but not the AR/ER ratio or AR expression alone, plays a prognostic role in metastatic BC patients who are treated with antiestrogen therapy [11]. We can contribute to the debate on the prognostic role of the ratio through a study assessing not only the AR/ER ratio, but also the AR/PgR and ER/PgR ratios in a subset of patients with disease progression where the meaning of these ratios have never been explored.

## 2. Methods

### 2.1. Case Series

For the present work we retrospectively analyzed a case series of patients consecutively enrolled from 2000 to 2008 at the Breast Unit of Morgagni-Pierantoni Hospital in Forlì. Eligible criteria were ≥18 years old, histological diagnosis of invasive BC, and a follow-up of at least 5 years. All patients had to have experienced distant disease relapse/progression. At least one specimen from the primary tumor and/or one from a metastasis had to be available for patients to be considered. The total number of patients analyzed was 159; primary tumor samples were available for all patients, while 24 had also a metastatic sample. All patients had to have hormone receptor expression data available. The Ethics Committee of IRST and AVR (Area Vasta Romagna) reviewed and approved the study protocol and patients provided written informed consent according to Italian privacy law.

### 2.2. Immunohistochemistry

The original hematoxylin and eosin stained specimens were reviewed by an expert pathologist to identify the most representative inclusion of tumor tissue for each patient. Neutral buffered formalin was used to fix tumor material obtained during surgery. Paraffin-embedded sections (four-micron thickness) were mounted on positive-charged slides for each sample (Bio Optica, Milan, Italy). Biomarker determinations were performed according to European Quality Assurance guidelines. Immunostaining for conventional biomarkers and AR expression was performed using the Ventana Benchmark XT staining system (Ventana Medical Systems, Tucson, AZ, USA) with the Optiview DAB Detection Kit (Ventana Medical Systems). ER, PgR, (Leica, Novocastra, Newcastle, UK), and AR (SP107 Cell Marque, Ventana Medical Systems) antibodies were used. For ER and PgR assessment, tissue sections were incubated for 60 minutes with antibodies diluted 1:80 and 1:40, respectively, in antibody diluents (Ventana Medical Systems). AR antibody, prediluted by the supplier, was used. Finally, all the sections were automatically counterstained for 16 minutes with hematoxylin II (Ventana Medical Systems). Biomarker expression was detected and semiquantitatively quantified as the percentage of immunopositive tumor cells on the total of tumor cells. Two independent observers evaluated all the samples and a discordance of >10% of positive cells was resolved by consensus after joint review using a multihead microscope. As clear guidelines for AR expression have not been available until now, we have not used a cut-off value. Given that we aimed to calculate the AR/ER and AR/PgR ratios, AR expression value was considered as a continuous variable (percentage of immunopositive tumor cells ranging 0-100%) and not dichotomous (positive/negative). As reported in our previous work, when ER or PgR were negative, the AR value was used instead of AR/ER and AR/PgR values [12].

### 2.3. Statistical Analysis

Overall survival (OS) was calculated as the time from the date of the start of first-line treatment for metastatic disease to the date of death from any cause or the date of the last follow-up visit. OS was estimated using the Kaplan-Meier method and compared with the log-rank test. Hazard ratios (HRs) and 95% confidence intervals (95% CI) were calculated using the Cox regression model. The optimal cut-off values were obtained from receiver operating characteristic (ROC) curve analysis at a median OS of 63 months. The concordance rate was calculated as the proportion of concordant cases with respect to the total number of patients. The two-sided exact binomial 95% confidence interval (95% CI) was estimated. All p values were based on two-sided testing and statistical analyses were performed using SAS statistical software version 9.4 (SAS Inc., Cary, NC, United States of America).

## 3. Results

We considered 159 BC patients with available primary tumor samples and data on AR and ER expression, of whom 125 patients had luminal tumor (defined as ER ≥1% and/or PgR ≥1% with any Ki67 or HER2 values). In order to calculate ratios we considered the 113 patients with AR and ER both ≥1%. Of these, survival data were available for 89 patients for whom we performed OS analyses. The patients' characteristics are reported in Table 1.

The median AR/ER ratio of the primary tumors in the luminal case series was 0.95 (range 0.06-95.00) while the median AR/PgR and ER/PgR ratios were 1.55 (range 0.06-95.00) and 1.60 (range 0.08-90.00) respectively (Table 2). The optimal cut-off values for AR/ER, AR/PgR, ER/PgR ratios to stratify patients according to prognosis were 0.95, 1.54, and 2, respectively. These values were obtained from receiver operating characteristic (ROC) curve analysis at a median OS of 63 months.

We evaluated the impact of the AR/ER, AR/PgR, ER/PgR ratios on OS. Hazard ratios (HRs) and 95% confidence intervals (95% CI) were calculated using the Cox regression model.

Median OS was longer for patients with AR/ER values < 0.95 (p value not significant) in primary tumors (Table 2).

OS was significantly shorter when the AR/PgR ratio was ≥ 1.54 for primary tumors (HR = 2.27; 95% CI 1.30-3.97;* p* = 0.004) (Table 2).

Similar results were obtained for ER/PgR ratio ≥ 2 in primary tumors where OS was significantly shorter (HR = 1.89; 95% CI 1.10-3.24;* p* = 0.021) (Table 2).

OS was better when AR/ER in the metastasis was ≥ 0.90 (HR = 0.09; 95% CI 0.01-0.70;* p* = 0.022) (Table 3). In addition, we assessed whether the difference in the AR/ER and AR/PgR ratios between primary tumor and metastasis influenced prognosis (Table 3).

Patients with a high AR/ER ratio in primary tumor that remained high in the metastasis had better prognosis in terms of OS (*p* = 0.011) (Table 3).

Analysis of the AR/ER ratio showed a concordance between primary tumors and metastases of 45.83% (95% CI 25.90-65.76) using 0.90 as cut-off value, whereas a concordance of 41.67% (95% CI 21.95-61.39) was observed for the AR/PgR ratio when the cut-off value was set at 0.96.

**Figure 1:**
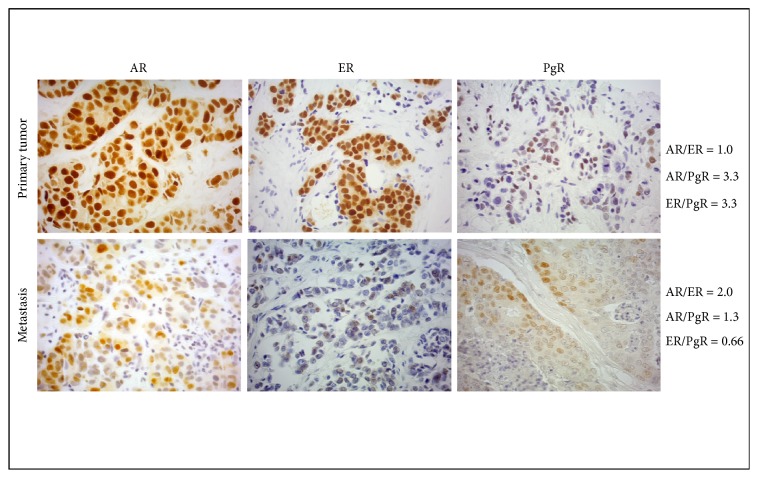
*Biomarker detection in breast cancer tissue*. Androgen receptor (AR), estrogen receptor (ER), and progesterone receptor (PgR) expression determined by immunohistochemistry (10X magnification) in primary tumor and metastasis from one patient, with relative AR/ER, AR/PgR, ER/PgR ratios calculated.

We report the immunohistochemical images of one patient for whom both primary tumor and metastasis specimens stained for AR, ER, and PgR were available. The AR/ER, AR/PgR, and ER/PgR ratios were calculated in each specimen to underline how they can change in different lesions from the same patient (Figure 1).

Another evaluation was done on the complete case series of 159 patients with any (positive or negative) values for ER, PgR, and AR. Of these, 104 patients had data on survival. Median ratios of AR/ER, AR/PgR, and ER/PgR were 0.95, 1.59, and 1.35, respectively (Table 4). OS was shorter when the ER/PgR ratio was ≥ 2 in primary tumors (HR= 1.90; 95% CI 1.14-3.17;* p* = 0.014). AR/ER and AR/PgR ratios did not predict OS (Table 4).

## 4. Discussion

We previously demonstrated an unfavorable prognostic role of the AR/ER ratio in different subsets of patients (i.e., patients with ductal carcinoma* in situ* of the breast), independently of treatment (*i.e.*, surgery alone or surgery plus radiotherapy) [12–14]. Similarly, Rangel et al. and Cochrane et al. reported a poorer prognosis when the AR/ER ratio was higher in the primary tumor of early BC patients [9, 10]. Then, considering that data have been reported in the literature only on the role of AR/ER ratio as unfavorable prognostic marker in primary tumor of early breast cancer patients, we performed a study in a different BC population, who presented disease relapse. We found in the luminal case series that the AR/ER ratio in primary tumor is not associated with prognosis and a significantly worse prognosis was observed when AR/PgR and ER/PgR were high. In both luminal and overall series the HRs went in the same direction for all the three ratios even if the statistically significant differences obtained were not the same. AR/PgR ratio was statistically different for the luminal case series, whereas ER/PgR ratios for both. PgR is an independent prognostic biomarker as previously demonstrated [15] and for this reason it may have a stronger prognostic impact than AR and ER in the ratios. Moreover an explanation of the cut-off values used for the ratios could be the different subset of patients analyzed. The finding of a risk of relapse 10-fold lower for patients with higher AR/ER values on metastases must be taken with caution, because it refers to a subgroup of patients whose AR/ER ratio on primary tumor differs from that of the entire case series, for the small sample size and the large confidence intervals. Patients who presented a high AR/ER ratio both in primary tumor and metastasis had a better prognosis.

## 5. Conclusion

Although our study was based on a small case series, it had the advantage of being able to compare primary tumor and metastatic samples from the same patients, which is fairly unusual in this setting. In conclusion, our findings indicate that a prospective study is needed to better clarify the role of AR/ER ratio in different BC settings (i.e., adjuvant and metastatic). The relation between AR and PgR has to be better understood even if our findings suggest that a high AR/PgR ratio in luminal tumors is prognostically unfavorable and could be used as an additional risk-stratification marker. The study of steroid hormone receptors and their relation with EMT process could lead to a more precise therapeutic approach.

## Figures and Tables

**Table 1:** Patients' characteristics.

	No. (%)
Total number	159 pts

Age	

<50	40 (25.2)
**≥**50	119 (74.8)

Tumor samples	

Primary	159 (100)
Metastasis	24 (15.1)
Both	24 (15.1)

T at diagnosis	

1	73 (50.0)
2	56 (38.4)
3	5 (3.4)
4	12 (8.2)
Unknown	13

N at diagnosis	

0	59 (41.8)
1	52 (36.9)
2	14 (9.9)
3	16 (11.4)
Unknown	18

M at diagnosis	

0	122 (76.7)
1	37 (23.3)

Grade	

1	6 (4.8)
2	48 (38.1)
3	72 (57.1)
Unknown	33

Adjuvant systemic therapy	

No adjuvant therapy	12 (7.5)
Chemotherapy	84 (52.8)
Hormone therapy	84 (52.8)
Both	58 (36.5)
Unknown	37 (23.3)

**Table 2:** Impact of AR/ER, AR/PgR, and ER/PgR ratios on OS in primary tumors of luminal BC patients (no. 89).

	*Median ratios (range)*			

*AR/ER*	0.95 (0.06 - 95.00)			

*AR/PgR*	1.55 (0.06 - 95.00)			

*ER/PgR*	1.60 (0.08 - 90.00)			

*OS according to best cut-off ratio*				
Median follow-up: 78 months (range 7 - 155)				

	*no. deaths/no. patients*	*Median OS (months)* *(95% CI)*	*HR* *(95% CI)*	*p*

Overall	55/89	63 (46-76)	-	-

*AR/ER*				

*<0.95*	28/47	64 (41-82)	1.00	

*≥0.95*	27/42	60 (42-83)	1.05 (0.62-1.78)	0.861

*AR/PgR*				

*<1.54*	21/39	82 (65-89)	1.00	

*≥1.54*	34/50	42 (34-56)	2.27 (1.30-3.97)	*0.004*

*ER/PgR*				

*<2.00*	25/40	82 (62-88)	1.00	

*≥2.00*	30/49	42 (34-64)	1.89 (1.10-3.24)	*0.021*

AR, androgen receptor; ER, estrogen receptor; PgR, progesterone receptor; OS, overall survival; HR, hazard ratio; CI, confidence interval; ND, not determinable; NR, not reached.

**Table 3:** Impact of AR/ER and AR/PgR ratios on OS in patients with AR and ER detected both in primary tumor and metastasis (no. 24).

*OS (HR) according to best cut-off of the ratios*				
		*Primary tumor*	*Metastasis*	

*AR/ER* *(cut-off 0.90)*		0.33 (95% CI: 0.08 – 1.36) p = 0.127	0.09 (95% CI: 0.01 - 0.70) p = 0.022	
*AR/PgR* *(cut-off 0.96)*		2.56 (95% CI: 0.56 – 11.72) p = 0.224	0.53 (95% CI: 0.18 – 1.59) p = 0.259	

*Median OS (months) according to AR/ER difference between primary tumor and metastasis*				

		*Metastasis*		

		<0.90	≥0.90	

*Primary tumor*	<0.90	32.4 (95% CI: 23.9 - NR)	36.4 (NR)	*p=0.011*
	≥0.90	42.2 (95% CI: 14.5 - 87.7)	92.0 (95% CI: 89.4 - NR)	

AR, androgen receptor; ER, estrogen receptor; PgR, progesterone receptor; OS, overall survival; HR, hazard ratio (AR/ER and AR/PgR ratios <cut-off are the reference category); CI, confidence interval; ND, not determinable; NR, not reached.

**Table 4:** Impact of the AR/ER, AR/PgR, and ER/PgR ratios, assessed on primary tumors, on OS in the overall series (no. 159).

*Median ratios (range)*				

*AR/ER*	0.95 (0 - 95)			

*AR/PgR*	1.59 (0 - 100)			

*ER/PgR*	1.35 (0 - 100)			

*OS according to best cut-off ratio*				
Median follow-up: 78 months (range 3-155)				

	*no. deaths/no. patients*	*Median OS (months)* *(95% CI)*	*HR (95% CI)*	*p*

Overall	63/104	62 (50-71)	-	-

*AR/ER*				

*<1.30*	53/88	63 (50 - 76)	1.00	

*≥1.30*	10/16	52 (23 - NR)	1.10 (0.56 - 2.16)	0.792

*AR/PgR*				

*<1.70*	31/51	66 (53 - 84)	1.00	

*≥1.70*	32/53	46 (36 - 65)	1.45 (0.88 - 2.39)	0.147

*ER/PgR*				

*<2.00*	27/49	81 (62 - 88)	1.00	

*≥2.00*	36/55	46 (36 - 60)	1.90 (1.14 - 3.17)	*0.014*

AR, androgen receptor; ER, estrogen receptor; PgR, progesterone receptor; OS, overall survival: HR, hazard ratio; CI, confidence interval; ND, not determinable; NR, not reached.

## Data Availability

The patient clinical and biological information and the dataset used for the analysis of this study are available from the corresponding author upon a reasonable request.
